# Use of Naltrexone for Patients With Stimulant Use Disorder in Malaysia: Protocol for a Retrospective Cohort Study

**DOI:** 10.2196/64101

**Published:** 2025-08-07

**Authors:** Nor Asiah Muhamad, Nur Hasnah Ma'amor, Muhammad Arif Muhamad Rasat, Tengku Puteri Nadiah Tengku Baharudin Shah, 'Izzah 'Athirah Rosli, Fatin Norhasny Leman, Nurul Hidayah Jamalluddin, Nurul Syazwani Misnan, Norliza Chemi, Norni Abdullah, Nurashikin Ibrahim, My Addiction Study group

**Affiliations:** 1 Sector for Evidence-based Healthcare National Institutes of Health Ministry of Health Selangor Malaysia; 2 Department of Psychiatry and Mental Health Sultanah Bahiyyah Hospital Ministry of Health Kedah Malaysia; 3 Department of Psychiatry and Mental Health Kajang Hospital Ministry of Health Selangor Malaysia; 4 Department of Psychiatry and Mental Health Tengku Ampuan Rahimah Hospital Ministry of Health Selangor Malaysia; 5 National Centre of Excellence for Mental Health Ministry of Health Cyberjaya Malaysia; 6 See Acknowledgments

**Keywords:** naltrexone, stimulant use disorder, abstinence, Malaysia, protocol

## Abstract

**Background:**

Naltrexone is an opioid receptor antagonist. Naltrexone is used to block the euphoric and sedative effects of drugs such as heroin, codeine, and morphine. The medication helps to bind and block opioid receptors to decrease opioid cravings. In Malaysia, naltrexone has been used for maintenance treatments for heroin and alcohol since 1996. However, since 2011, naltrexone has been used as an off-label stimulant use disorder (StUD) treatment to achieve abstinence.

**Objective:**

This study aims to determine the abstinence among StUD and non-StUD patients treated and without naltrexone.

**Methods:**

We will conduct a retrospective cohort study on the effect of naltrexone or treatment as usual (TAU) by examining the data for both StUD and non-StUD patients. We will use patients’ clinical records from the hospital registry. All adult patients (aged 18-65 years) diagnosed with StUD or other substance use disorders who were treated with naltrexone and standard care from January 1, 2011, to December 31, 2023, will be screened. All StUD and non-StUD patients who were offered the naltrexone treatment or TAU at the beginning of treatment will be recruited. All data will be extracted using a standardized data extraction form. Descriptive analysis will be performed to describe the distribution of patient characteristics, sociodemographic profiles, and percentages of abstinence and treatment retention. We will conduct univariable analysis to determine the association of stimulant abstinence and treatment retention between naltrexone and TAU among both StUD and non-StUD patients. All significant independent variables will be further analyzed using a cross-sectional time series method for categorical variables.

**Results:**

Recruitment began in July 2025. Data analysis will begin after completing data collection, planned for January 2026.

**Conclusions:**

The expected main outcome of this study is to observe the significant associations of stimulant use abstinence and treatment retention between TAU and naltrexone among StUD and non-StUD patients. The findings from this study may provide preliminary evidence regarding the use of naltrexone in treating StUD. Currently, there is no specific medication to treat amphetamine or methamphetamine use disorder. The effect of naltrexone with psychosocial interventions for StUD is unclear. Public health approaches recognize the multifaceted nature of substance misuse and focus on addressing the myriad individual, environmental, and social factors that contribute to StUD.

**International Registered Report Identifier (IRRID):**

PRR1-10.2196/64101

## Introduction

Stimulant use disorder (StUD) is a global epidemic characterized by continued use of stimulants that leads to clinically significant impairment or distress, resulting in tens of thousands of deaths annually [1]. Cocaine, amphetamine-type substances, and other prescribed drugs [2,3] are categorized as stimulant drugs. Amphetamine-type stimulants (ATSs) such as 3,4-methylenedioxymethamphetamine (MDMA or ecstasy) and methamphetamine are currently reported as the second most commonly used drug type, surpassing cocaine and heroin users. Over one-third of amphetamine users are from East and Southeast Asia [3,4]. A report from Malaysia’s National Anti-Drug Agency’s 2020 data stated that ATSs were the most used drug in Malaysia [5]. This observed trend was accompanied by the concerning fact that StUD results in several detrimental effects associated with not only medical conditions, such as strokes, aneurysm rupture, and seizures, but also violent crime cases in Malaysia [6-11].

Thus far, treatment for amphetamine users in emergency departments and psychiatric clinics has focused on behavioral interventions and management of acute and short-term consequences of amphetamine dependence, such as withdrawal symptoms, amphetamine-induced psychosis, and associated depression [12]. Other treatments for amphetamine use disorder include acupuncture and pharmaceutical interventions such as antidepressants, antipsychotics, and anticonvulsants [12]. Besides, nonpharmacological treatments such as cognitive behavioral therapy, contingency management, community reinforcement (incentivize patients to remain engaged in treatment in community-based substance use or mental health clinics), and relapse prevention have also been introduced [13].

Naltrexone is an opioid receptor antagonist. Naltrexone inhibits the euphoric and sedative effects of substances like heroin, codeine, and morphine by binding and blocking opioid receptors. This decreases patients’ craving and tolerance to opioids [14-17]. Few studies have proposed naltrexone as a treatment for StUD and demonstrated a significant effect in reducing the consumption and craving for amphetamine [18-20]. Besides, an acute dose of naltrexone blunted the subjective effects of amphetamine under experimental laboratory conditions, for example, decreasing the percentage of amphetamine-positive urine samples in patients with chronic amphetamine dependence. This indicates the possibility of naltrexone pharmacotherapeutic treatment for amphetamine dependence [18-21]. In contrast, research by Schottenfeld et al [22] stated that the effect of naltrexone in patients with heroin use disorder was not statistically different from a placebo when compared to buprenorphine. The aforementioned studies [18,20] have been included in a systematic review in addition to other studies demonstrating inconsistent findings [23]. Despite extensive research elucidating the neurobiological effects of amphetamines, effective pharmacotherapies remain elusive [24]. However, this review does not include empirical investigations into associations between nonstimulant substance use disorders and neurological traits.

In Malaysia, naltrexone has been used for maintenance treatment of heroin dependence since the late 1990s, with studies supporting both its clinical effectiveness and cost-efficiency in the local context [22,25]. Although its use for alcohol and stimulant dependence has been explored internationally [26,27], evidence specific to Malaysia remains limited. Since 2011, naltrexone has been used off-label as a treatment option for StUD [23]. However, no study to date has examined the association between naltrexone and abstinence outcomes or treatment retention among patients with StUD in the Malaysian setting. Therefore, this study will be conducted to test the hypothesis that naltrexone can be effective in achieving abstinence among patients with StUD and that naltrexone is effective as part of a treatment strategy for stimulant use. Furthermore, this study will also support clinicians by providing the best practice for patients with StUD. These efforts will require precision and accuracy in our translation of the literature. Thus, this study will also describe the rationale and targets for pharmacotherapies for ATS dependence, identify other emerging pharmacogenetic data, and propose directions for future work.

## Methods

### Study Design

We will conduct a retrospective cohort study by viewing the patients’ files to measure the effect of naltrexone compared to treatment as usual (TAU). The researchers, clinicians, nurses, and medical assistants who will be involved in this study will be trained for 3-4 sessions before data collection. Patients will be identified by the trained hospital staff (clinicians, nurses, and medical assistants). Adult patients aged 18-65 years diagnosed with StUD and non-StUD (ie, those with other substance use disorders, such as alcohol and smoking) treated with or without naltrexone from January 1, 2011, to December 31, 2023, will be recruited. We will collect the data using a standardized data extraction form or clinical report form (Multimedia Appendix 1), which will be piloted with 30 patients in this study. We will extract the data from the patients’ clinical records. The data will be anonymized prior to data extraction. Data will be collected through web-based Google Forms by the trained researcher and hospital staff. We will include all patients with StUD and non-StUD who were offered the option of naltrexone treatment or TAU. We will exclude the patients who were younger than 18 years.

### Study Locations

The study locations will be randomly selected using stratified random sampling based on the zones in Malaysia. Hence, we will identify a total of 15 government hospitals across Malaysia with addiction specialists.

### Sample Size Calculation

We have calculated the sample size using a two-proportion formula using Power and Sample Size Calculation (version 3.1.2; WD Dupont and WD Plummer) for both StUD and non-StUD patients. A previous study indicated that the probability of stimulant abstinence among patients with StUD was 0.5 when treated with a placebo and 0.65 when treated with naltrexone [7]. A minimum of 113 patients with StUD receiving TAU with naltrexone and 339 patients with StUD receiving TAU without naltrexone are needed to be able to reject the null hypothesis, where abstinence for StUD with and without naltrexone is equal, with the power set at 80%. The type 1 error probability associated with the test of this null hypothesis is 0.05. We will use an uncorrected χ^2^ statistic to evaluate this null hypothesis. Considering 20% of data being incomplete or missing, a total of 136 patients with StUD treated with naltrexone and 136 patients with StUD treated without naltrexone are needed for this study.

The sample size calculation for the non-StUD group referred to a previous study that indicated a 0.23 probability for stimulant abstinence among non-StUD patients treated with a placebo and 0.17 for non-StUD patients treated without naltrexone [11]. A similar formula, accounting for type 1 error probability and 20% incomplete or missing data, was used for the calculation. Therefore, both non-StUD groups need a minimum total of 490 non-StUD patients, with 245 treated with naltrexone and 245 treated without naltrexone.

### Study Variables

The following definitions of the cohorts and related terms will be used during data collection:

- Cohort 1: patients diagnosed with StUD and treated with naltrexone and TAU
- Cohort 2: patients diagnosed with StUD and treated with TAU
- Cohort 3: patients diagnosed without StUD and treated with naltrexone and TAU
- Cohort 4: patients diagnosed without StUD and treated with TAU
- Abstinence: the state of being abstinent from a substance (drug free), specifically stimulants (ATSs)
- Treatment retention: Patients with StUD continue naltrexone and TAU

### Research Tool

The data extraction form will be divided into five sections: (1) sociodemographic profile, (2) medical and clinical background, (3) stimulant use (dosage, duration, and routes of administration), (4) StUD or non-StUD treatment, and (5) urine screening.

#### Section 1: Sociodemographic Profile

This section will collect information about the sociodemographic profile of the patients, such as gender, age, ethnicity, marital status, education, occupation, and current housing area.

#### Section 2: Medical and Clinical Background

This section will collect information on the patients’ medical and clinical history, including the hospital where treatments are received; history of psychiatric illness; history of hypertension, diabetes, cardiovascular disease, renal disease, or other disease; and history of any behavioral addiction such as gambling disorder or internet addiction.

#### Section 3: Stimulant Use

This section will collect information on the use of stimulants by the patients. This includes the name of the stimulants, stimulant dosage, routes of administration, frequency of use, age (in years) when stimulant use started, reason for taking stimulant, and duration of use.

#### Section 4: StUD or Non-StUD Treatment

This section will collect information on the treatment received by patients with StUD and whether the patient received naltrexone. Other information includes the start date of treatment, doses of treatment received, duration of treatment received, and reason for stopping treatment, if applicable.

#### Section 5: Urine Screening

This section collects the results of urine drug tests for amphetamine and methamphetamine to study the abstinence from stimulants among patients with StUD treated as usual with and without naltrexone. A similar method will be applied to the non-StUD group. Records of positive and negative results from urine tests for stimulant metabolites will be obtained every 3 months for 1 year (admission, month 3, month 6, month 9, and month 12).

### Statistical Analyses

We will analyze the data separately in a few categories regarding abstinence from substances (drug free) for 1 month, 3 months, 6 months, 9 months, and 12 months. A similar timeline will be applied when investigating treatment retention for both StUD and non-StUD patients while receiving treatment with naltrexone and TAU. The patients will be separated into 4 groups for analysis: (1) patients with StUD treated with naltrexone, (2) patients with StUD treated without naltrexone, (3) non-StUD patients treated with naltrexone, and (4) non-StUD patients treated without naltrexone. If there is any missing data, we will try to search for the patients’ old notes or files, or ask clinicians to contact the patients to obtain the data.

We will analyze the data using Stata software (version 17; StataCorp), along with data cleaning and data verification. Before conducting analyses, data will be verified for errors, statistical assumptions, and potential confounding variables to examine as covariates. Descriptive analysis will be performed to describe the characteristics of the patients, sociodemographic profiles, and percentages of abstinence and treatment retention. Continuous variables will be summarized using means (SDs). Categorical variables will be presented as counts (n) and percentages (%). Univariable analysis will be performed using the χ^2^ test to determine the association of abstinence from stimulants and treatment retention between the naltrexone and TAU groups among StUD and non-StUD patients. We will perform cross-sectional time series analysis for any significant associations (*P*<.05) in the univariable analysis based on the time points of 1 month, 3 months, 6 months, and 12 months.

### Ethical Considerations

This study will be funded by the National Centre of Excellence for Mental Health, Ministry of Health, as a mental health study. We have registered this study with the National Medical Research Register, Ministry of Health Malaysia, with the registration number NMMR ID-24-00626-TBA. We have received ethics approval from the Medical Research & Ethics Committee, Ministry of Health, Malaysia with the reference number NMRR ID-24-00626-TBA (IIR). The Medical Research & Ethics Committee allows the use of secondary data if the data is anonymized; therefore, no informed consent was needed. Patients’ IDs will be anonymized or deidentified, and new IDs will be given by the researcher that will be used in place of their name or other identifying information. All data will be kept in secure, password-protected files. Although we plan to make the data publicly available for reanalysis, we will carefully deidentify the data first. For publications or presentations of the results, any information that could identify the participant will not be provided.

## Results

Recruitment began in July 2025. Data analysis will begin after data collection, planned for January 2026. For a full study schedule, see Multimedia Appendix 2. We plan to disseminate the results from this study at an international conference or publish in an international journal.

## Discussion

### Expected Findings

Significant associations in StUD abstinence and treatment retention between TAU and naltrexone among StUD and non-StUD patients are expected to be observed. The findings from this study may provide preliminary evidence to update the clinical practice guideline on the use of naltrexone in treating stimulant misuse.

Currently, no medication is approved for the treatment of amphetamine or methamphetamine use disorder [13], and the effects of naltrexone combined with psychosocial interventions for StUD remain unclear [21]. Clinically, naltrexone’s role in improving abstinence has been observed in patients with alcohol use disorder [26], smoking dependence [27], and amphetamine dependence [19], although contrasting findings have also been reported [23]. However, no local studies in Malaysia have examined the role of naltrexone in treating StUD, despite the significant social impact substance misuse has on communities. Naltrexone is currently used off-label for both StUD and non-StUD populations. Therefore, this study aims to provide evidence on the effectiveness of naltrexone in supporting abstinence among Malaysian patients with StUD and non-StUD, ultimately contributing to best clinical practices and bridging the local research gap on this treatment approach.

This study will also highlight the need for public health approaches to recognize the multifaceted nature of substance misuse and focus on addressing the myriad individual, environmental, and social factors that contribute to StUD. Since prevention programs address risk and protective factors that are common in a range of behavioral problems, a naltrexone program for the treatment of StUD will produce positive outcomes not only in preventing drug misuse but also in reducing aggression, early behavioral change, and impaired driving, and improving mental health and educational outcomes.

### Limitations

There are a few limitations that should be considered for this study. As this study will collect the data from the patients’ files, the patients’ information regarding abstinence and treatment retention may be different, and therefore, we will separate the analysis into a few categories: 1 month, 3 months, 6 months, 9 months, and 12 months to overcome this problem. Some of the data may not be complete, such as history of other psychiatric diseases or health issues.

### Dissemination

This study is intended for publication in a psychiatric journal such as *JAMA Psychiatry*, *World Psychiatry*, or *BMC Psychiatry*. We may also publish in high-impact journals that are suitable for our topic, such as *BMJ*, *PLoS*, or a JMIR Publications’ journal. A few findings will be presented at both local and international conferences. The preliminary findings will be presented to the Psychiatry Clinical Practice, Ministry of Health, Malaysia. This study will also help clinicians decide the best treatment for the patients and might be used as a reference when developing clinical practice guidelines. We will also produce plain language summaries that will be shared with the public through social media and health care websites.

## Appendix

Multimedia Appendix 1 Data extraction form.

Multimedia Appendix 2 Timeline.

Multimedia Appendix 3 STROBE (Strengthening the Reporting of Observational Studies in Epidemiology) checklist.

## Abbreviations

ATS: amphetamine-type stimulant
MDMA: 3,4-methylenedioxymethamphetamine
StUD: stimulant use disorder
TAU: treatment as usual

## Data Availability

The data will be provided with the final manuscript in an Excel sheet format.
