# Supplementary material for: Use of Naltrexone for Patients With Stimulant Use Disorder in Malaysia: Protocol for a Retrospective Cohort Study
Source: JMIR Res Protoc. 2025 Aug 7;14:e64101. doi: 10.2196/64101 (PMC12371278; doi:10.2196/64101)
Supplement: Multimedia Appendix 1 [file resprot_v14i1e64101_app1.docx]

Part A: Demographic data

# Subject ID

2. Hospital *

## Mark only one oval.


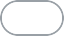
 Hospital Tuanku Ja’afar, Seremban, Negeri Sembilan (HTJ)
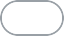
 Hospital Kuala Lumpur (HKL)


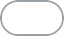
 Hospital Tengku Ampuan Rahimah, Klang, Selangor (HTAR)
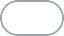
 Hospital Kajang, Selangor (HKJ)


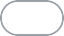
 Hospital Melaka (HM)


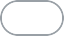
 Hospital Sultanah Bahiyyah, Kedah (HSB)
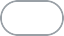
 Hospital Permai, Johor (HP)


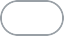
 Hospital Tuanku Fauziah, Perlis (HTF)
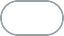
 Hospital Queen Elizabeth, Sabah (HQE)
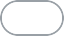
 Hospital Umum Sarawak, Sarawak (HUS)


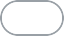
 Hospital Raja Perempuan Zainab II, Kelantan (HRPZ)
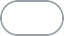
 Hospital Sultanah Nur Zahirah, Terengganu (HSNZ)
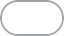
 Hospital Tuanku Ampuan Afzan, Pahang (HTAA)
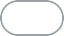
 Hospital Raja Permaisuri Bainun, Perak (HRPB)
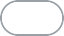
 Hospital Mesra Bukit Padang, Sabah (HMBP)

# 3. Age (years) *

4. Gender *

## Mark only one oval.


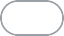
 Male
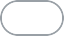
 Female

# 5. Ethnicity *

## Mark only one oval.


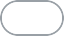
 Malay
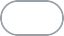
 Chinese
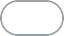
 Indian
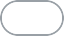
 Others

# If others, please specify

6. Education level *

## Mark only one oval.


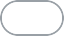
 No formal education (no official certification from the government, sekolah pondok)


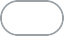
 Primary
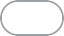
 Secondary
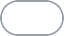
 Tertiary
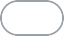
 Not stated

# 7. Occupation *

## Mark only one oval.


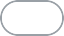
 Employed (eg : small business)


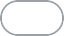
 Unemployed (eg : retired, stay at home)
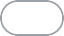
 Student


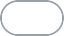
 Not stated

# 8. Current housing Area *

## Mark only one oval.


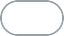
 Urban
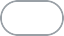
 Rural
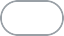
 Not stated

# 9. Marital status *

## Mark only one oval.


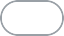
 Single
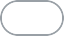
 Married
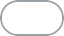
 Divorcee


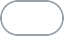
 In relationship
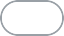
 Not stated

Part B: Clinical information

# 1. Co-occurring Diagnosis *

*Check all that apply.*


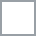
 Psychotic disorder (schizophrenia, brief psychotic disorder, schizophreniform)


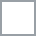
 Mood/affective disorder (major depression disorder (MDD), persistent depressive, bipolar)


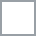
 Anxiety related disorder (anxiety disorder, generalized anxiety disorder, social anxiety disorder, panic disorder)


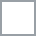
 Organic related disorder (organic brain syndrome, psychotic disorder secondary to another medical condition, depressive disorder secondary to medical condition)


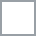
 Neurodevelopmental disorder (intellectual disability, learning disorder, ADHD, Autism spectrum disorder)

Other:

# If others, please stated

1. Type of Substances

*Check all that apply.*

Yes No

Opiiattes (herroiin,, morrphiine,, codaiine,, ttrramadoll,, petthiidiine)

Allcoholl

Cannabiis (marriijjuana)

Sttiimullantts (metthamphettamiine,, amphettamiine,, cocaiine,, iice,, syabu)

Sedattiive Hypnottiic (benzodiiazepiines,, erriimiin 5)

Kettum (krrattom)

Halllluciinogens (LSD,, Phencyclliidiine,, kettamiine)

IInhallantts (gam) Niicottiine

New Psychoacttiive Substtances/NPS (syntthettiic drrugs/mushrroom/IIlllliiciitt drrugs)

Ottherrs

# If others, please state

1. Behavioral addiction *

## Mark only one oval.


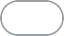
 Yes
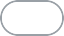
 No

# If yes, please specify

*Check all that apply.*


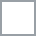
 Gambling disorder
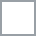
 Internet addiction


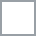
 Problematic sexual behavior (pornography addiction, masturbation addiction)
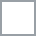
 Gaming addiction

Other:

# If others, please clarify

1. History of medical illness

*Check all that apply.*


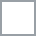
 Hypertension
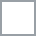
 Diabetes


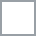
 Heart Disease
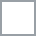
 Renal Disease
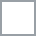
 Others


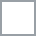
 None

# If others, please state

Part C: Stimulant Use

# 1. Type of stimulants use

*Check all that apply.*


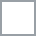
 Amphetamine


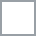
 Methamphetamine (eg: pil kuda, syabu, ICE, batu)
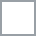
 MDMA (eg: ecstasy)


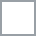
 Cocaine


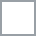
 New Psychoactive Substances/ NPS (eg: synthetic drugs/mushroom)

Other:

# If others, please stated.

2. Route of administration

*Check all that apply.*


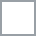
 Inhalation


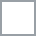
 Oral ingestion
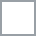
 Injection


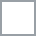
 Not stated

# 3. Frequency of use during initiation of NT/other treatment

## Mark only one oval.


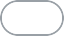
 Daily use (4 or more times/week)
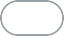
 2 - 3 times/week


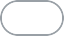
 2 -4 times/month
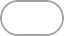
 Monthly or less
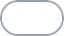
 Not stated


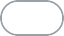
 None

# 4. Age of first use of stimulant (years)

5. Duration of taking the stimulant (years)

SUD Treatment

# 1. Type of SUD treatment received *

## Mark only one oval.


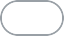
 TAU only


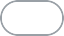
 TAU with naltrexone only


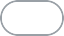
 TAU with other psychotropics (please specify)


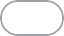
 TAU with naltrexone and other psychotropics (please specify)

Treatment with other psychotropics (please specify)

2. Has Naltrexone been used in this hospital? *

If No, proceed to urine screening section.

## Mark only one oval.


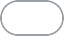
 Yes


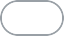
 No *Skip to question 33*

# 3. Starting date of receiving naltrexone treatment

*Example: January 7, 2019*

4. Dose of naltrexone treatment

5. Duration of naltrexone treatment (range for example April 2020 - April 2021)

6. Reason of dropping out

*If patients stop receiving treatment in the middle of the 1 year study*

Urine screening (measure of abstinence)

# 1. Urine drug test for stimulant's metabolite *

*Check all that apply.*

Positive Negative/traces Not

available

Enttrrance on Admiissiion

Montth 3

Montth 6

Montth 9

Montth 12

# 2. Positive stimulant's metabolite

*If negative, ignore this question*

# 3. Follow-up attendance to receive treatment *

*Check all that apply.*

Yes No

Enttrrance on Admiissiion

Montth 3
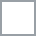


Montth 6
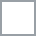


Montth 9
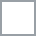


Montth 12
