# Supplementary material for: Use of Naltrexone for Patients With Stimulant Use Disorder in Malaysia: Protocol for a Retrospective Cohort Study
Source: JMIR Res Protoc. 2025 Aug 7;14:e64101. doi: 10.2196/64101 (PMC12371278; doi:10.2196/64101)
Supplement: Multimedia Appendix 2 [file resprot_v14i1e64101_app2.docx]

**Gantt Chart**

| **Year** | **2024** | | | | **2025** | | | | | | | | | | | | **2026** | | | | |
| --- | --- | --- | --- | --- | --- | --- | --- | --- | --- | --- | --- | --- | --- | --- | --- | --- | --- | --- | --- | --- | --- |
| **Month** | **Sept** | **Oct** | **Nov** | **Dec** | **Jan** | **Feb** | **Mac** | **Apr** | **May** | **Jun** | **July** | **Aug** | **Sept** | **Oct** | **Nov** | **Dec** | **Jan** | **Feb** | **Mac** | **Apr** | **Mei** |
| **Activities** |  |  |  |  |  |  |  |  |  |  |  |  |  |  |  |  |  |  |  |  |  |
| **Preparation of study proposal** | **x** | **x** | **x** |  |  |  |  |  |  |  |  |  |  |  |  |  |  |  |  |  |  |
| **Approach study site** |  |  |  | **x** | **x** | **x** |  |  |  |  |  |  |  |  |  |  |  |  |  |  |  |
| **Develop of clinical research form** |  |  |  |  | **x** | **x** | **x** | **x** | **x** | **x** |  |  |  |  |  |  |  |  |  |  |  |
| **Data collection** |  |  |  |  |  |  |  |  |  |  | **x** | **x** | **x** | **x** | **x** | **x** |  |  |  |  |  |
| **Data entry** |  |  |  |  |  |  |  |  |  |  | **x** | **x** | **x** | **x** | **x** | **x** |  |  |  |  |  |
| **Data cleaning** |  |  |  |  |  |  |  |  |  |  | **x** | **x** | **x** | **x** | **x** | **x** | **x** |  |  |  |  |
| **Data analysis** |  |  |  |  |  |  |  |  |  |  |  |  |  | **x** | **x** | **x** | **x** | **x** | **x** | **x** |  |
| **Report writing** |  |  |  |  |  |  |  |  |  |  |  |  |  |  |  |  | **x** | **x** | **x** | **x** | **x** |
| **Publish manuscript** |  |  |  |  |  |  |  |  |  |  |  |  |  |  |  |  |  |  |  |  | **x** |
